# Experimental infection of lambs with C and S-type strains of *Mycobacterium avium* subspecies *paratuberculosis*: immunological and pathological findings

**DOI:** 10.1186/1297-9716-45-5

**Published:** 2014-01-16

**Authors:** Miguel Fernández, Julio Benavides, Iker A Sevilla, Miguel Fuertes, Pablo Castaño, Laetitia Delgado, J Francisco García Marín, Joseba M Garrido, M Carmen Ferreras, Valentín Pérez

**Affiliations:** 1 Departamento de Sanidad Animal, Instituto de Ganadería de Montaña (CSIC-ULE), Facultad de Veterinaria, Universidad de León, Campus de Vegazana s/n, León 24071, Spain; 2 Departamento de Sanidad Animal, NEIKER-Tecnalia, Berreaga 1, Derio Bizkaia 48160, Spain

## Abstract

The two main genotypes of recognized isolates of *Mycobacterium avium* subsp. *paratuberculosis* (*Map)* are cattle (C) and sheep (S) strains. An experimental infection was conducted to establish the effect of *Map* strain on the pathogenesis of ovine paratuberculosis. Twenty-four out of thirty 1.5-month-old Assaf lambs were divided into 4 groups of 6 and infected orally with three low passage field isolates, two of S- (22G and the pigmented Ovicap49) and one of C– (764) type, and the reference K-10 strain (C type). The remaining six animals were unchallenged controls. Animals were euthanized at 150 and 390 days post-infection (dpi). Throughout the experiment, the peripheral immune response was assessed and histological and molecular (PCR) studies were conducted on samples of intestine and related lymphoid tissue. Specific antibody and IFN-*γ* production was significantly higher in animals infected with the C strains, while no consistent IFN- γ responses were observed in the S-type strain infected groups. A positive intradermal skin test response was detected in all infected groups. Lambs infected with S-type strains had granulomatous lesions restricted to the lymphoid tissue with no differences in the lesion intensity over time. In both C–type strain groups, lesions were more severe at 150 dpi while at 390 dpi lesions, characterized by well-demarcated granulomas with fibrosis, decreased in severity. Only infected lambs were positive to PCR. These results suggest that the strain of *Map* has a strong influence over the immune and pathological responses developed by the host. Lesions induced by C–type strains in lambs show a regressive character and tend to decrease as the infection progresses.

## Introduction

Paratuberculosis or Johne’s disease, caused by *Mycobacterium avium* subspecies *paratuberculosis* (*Map*), is a chronic infection of domestic and wild ruminants characterized by granulomatous enteritis and lymphadenitis. It occurs worldwide and causes high economic losses to domestic livestock. Clinical disease results in a progressive loss of weight, usually with chronic diarrhoea, and eventual death of the infected animals.

Pathogenesis of paratuberculosis is still poorly understood. It is assumed that animals become infected early in life and can develop a variety of responses: resistance to infection, an asymptomatic status in which animals remain subclinically infected for life, or clinical disease [1-3]. Different pathological responses have also been detected among infected animals, both in natural and experimental cases depending on the intensity, location, cellular types and number of acid-fast bacilli present in the granulomas [4-6]. Briefly, these lesions are divided into *focal* forms, characterized by the presence of small granulomas restricted to the Peyer’s patches; *multifocal* lesions with granulomas present in the intestinal mucosa regardless of its association with lymphoid tissue, and *diffuse* forms, related to clinical signs, characterized by a widespread and diffuse granulomatous enteritis.

It has been suggested that these differences could be related to the immune response mounted by the host in such a way that a great number of asymptomatic animals with focal or multifocal lesions show an intense cell-mediated immune response, while the detection of the humoral immunity would be related to clinical disease [7-9]. There is also evidence suggesting that these variations in pathogenesis could be due to differences in the microorganism [10-12].

The existence of distinct strains of *Map* based on phenotypic differences has been recognised for some time [13]. Recently the use of IS*900* restriction fragment length polymorphism (RFLP) and IS*1311* PCR-restriction enzyme analysis (PCR-REA) methods, has led to the classification of *Map* strains into two main genotypes: sheep or ovine isolates (also called “S-type” or “type-I and III”) and cattle or bovine isolates (also called “C-type” or “type II”) [14-16]. Although not common, cross-species infections have been documented [17-19]. Besides the genotypic distinctions between C and S strains of *Map*, phenotypic differences have been found in in vitro experiments, where infected macrophages exhibit different inflammatory responses depending on the type of isolate [11,20,21]. Variations in the peripheral immune response have also been reported after the infection of sheep with C and S strains of *Map*[10]. Furthermore, in contrast to C-type isolates, the growth of S-type *Map* strains in culture media is slower and more fastidious [19,22]. The existence of pigmented isolates among S-type strains is also well documented [13,16].

Concerning the role of *Map* strains in the development of the different pathological responses, a previous study [23] showed that S-type strains cause diffuse and more severe lesions than C strains in experimentally infected lambs. However, in that study, the ovine *Map* inoculum was an intestinal mucosa homogenate from a field case of paratuberculosis and no molecularly typified strains were employed. Furthermore, the presence of lesions was only assessed at 150 days post-infection (dpi).

The aim of this study was to evaluate the influence of the different strains of *Map* on the pathogenesis of paratuberculosis, through the evaluation of lesion development and peripheral immune responses in lambs experimentally infected with molecularly typed C and S strains obtained from pure cultures.

## Materials and methods

### Inocula preparation

The strains used for the challenge were the reference strain of *Map* K-10 (ATCC® BAA-968™) and three low-passage field isolates maintained as glycerol stocks at -80 °C obtained from different species and samples. The latter strains represent the most widespread C and S genotypes found in Spain as previously reported [24]. More detailed information on the strains is shown in Table 1. All strains were propagated in Middlebrook 7H9 broth [25] supplemented with OADC (oleic acid, albumin, dextrose, catalase) enrichment (Becton Dickinson and Company, MD, USA), Tween 80 (Panreac Quimica SA, Barcelona, Spain), glycerol and mycobactin J (Allied Monitor, Inc., Fayette, MO, USA). After 4–5 weeks at 37 °C, cultures were harvested by centrifugation at 2800 × *g* for 15 min. Bacterial pellets were washed twice in phosphate buffered saline (PBS), resuspended in PBS and the presence of clumps minimized by making the liquid flow up and down through a fine needle (26G3/8) several times. Turbidity (McFarland units) of suspensions was measured using a Densimat (bioMérieux, Marcy l’Etoile, France). Expected cell concentration was estimated considering one McFarland unit as 10^8^ cells/mL according to a previous study [26] but taking into account that this equivalence could be one log lower as reported elsewhere [25]. Suspensions were adjusted to 2 × 10^9^ cells/mL with PBS and each challenge whole-dose prepared using one mL of these suspensions as explained below.

**Table 1:** Origin of the strains used in the infection

**Strain ID**	**Host**	**Breed**	**Isolated from**	**Location**	**IS1311 PCR-REA**	***Sna*** **BI- *Spe *** **I PFGE**
22G	Sheep	Latxa	intestinal tissue	Gipuzkoa, Spain	S	69-50 Type III
Ovicap49^a^	Sheep	Latxa	intestinal tissue	Navarra, Spain	S	57-57 Type III
764	Cattle	Holstein	feces	Bizkaia, Spain	C	2-1 Type II
K-10	Cattle		feces	USA	C	1-1 Type II

^a^Pigmented strain.

Ten-fold serial dilutions were prepared and plated onto agar-solidified 7H9 with OADC, glycerol and mycobactin J in quadruplicate to assess the number of colony forming units (CFU) per mL in the inocula. Since bacteria were administered to animals in several aliquots and on separate days during 2 weeks, the unused aliquots of diluted inocula were kept at 4 °C until required. In order to assess any potential loss of viability during this time, the plating procedure was repeated the last challenge day when all aliquots were administered. The material used to assess the potential reduction in CFU/mL numbers was a separate aliquot prepared and kept under the same conditions as those of the administered doses.

### Experimental animals

A total of thirty 1.5-month-old lambs of the Assaf breed were used in this study. They were randomly selected from a flock in which no clinical cases of paratuberculosis had been reported in the last 5 years. Antibody ELISA and IFN-γ release test was performed in all the dams of the lambs, and all were negative to both assays.

After a period of adaptation in the experimental facilities of the “Instituto de Ganadería de Montaña CSIC-ULE”, the lambs were allocated in separate pens and randomly divided into the following five groups, each composed of six lambs, according to the strain of *Map* inoculated: 22G and Ovicap49 sheep strains; 764 and K-10 cattle strains and a fifth group of uninfected control animals challenged with saline solution. All the animals followed a diet based on fed grass hay *ad libitum* and a conventional compound feed appropriate for each age.

### Experimental design

The experimental procedures carried out in this study were performed in accordance with Spanish Royal Decree 1201/2005 for the protection of animals used for experimental and other scientific purposes, and were approved by the “Instituto de Ganadería de Montaña CSIC-ULE” Animal Ethics Committee.

Each experimentally infected lamb was orally inoculated using an automatic syringe with a total amount of 2 2 × 10^9^ mycobacteria diluted in 40 mL of PBS that was divided into four doses of 10 mL, administered at 3-day intervals.

On day 150 after infection, two lambs from each group were humanely culled by the intravenous injection of a veterinary euthanasia drug (T61®, Intervet, Salamanca, Spain), followed by exsanguination. The remaining animals were killed at 390 dpi.

Blood samples were collected from the jugular vein into 10 mL evacuated tubes (Venoject®, Terumo Europe N.V., Leuven, Belgium) containing lithium heparin or without anticoagulant for IFN-γ and antibody (Ab) determination studies respectively. Blood samples were taken at monthly intervals from day 0 up to 390 dpi.

### Cell mediated immune response determination

#### Interferon-γ (IFN-γ) release assay (IGRA)

For the IFN-γ test, whole blood samples taken in heparinized tubes were used. They were always processed within 3 h from the time of collection. Two separate aliquots of 1.5 mL blood were mixed with either 100 μL of sterile PBS (negative control) or an avian purified protein derivative (PPD) Ag (CZ Veterinaria, Porriño, Spain) at a final concentration of 30 μg/mL. Whole-blood cultures were incubated for 20 h at 37 °C in a humidified atmosphere. The tubes were then centrifuged and plasma supernatant was removed and frozen at -20 °C until required. Plasma samples were then assayed in duplicate for the IFN-γ determination using a commercial immunoassay kit (“BOVIGAM®” *Mycobacterium bovis* Gamma Interferon Test Kit for cattle, Prionics AG, Switzerland) that has been widely used for testing ovine samples [9,10,23,27], according to the manufacturer’s instructions. For avoiding inter-plate variations, all the O.D. values were adjusted by dividing the sample O.D. minus the negative control O.D., from each plate. Once the raw values were standardized, the results were expressed as a quotient between the mean O.D. of the avian PPD-stimulated plasma and the mean O.D. of the sample plasma incubated with PBS. An animal was considered as positive when the quotient was higher than 2 [9,27].

#### Single intradermal skin test (IDT)

One month before sampling (120 and 360 dpi), all the lambs were injected intradermally in the skin fold of the tail with 0.1 mL of avian PPD (CZ Veterinaria, Porriño, Spain) at a 0.5 mg/mL concentration. Skin-fold thickness was measured at the injection site with a calliper before injection and 72 h later. The results were expressed as the increase in millimeter of skin thickness. When used for diagnostic purposes, an animal was considered as positive when the increase in skin thickness was ≥ than 2 mm [9].

#### Humoral immune response determination by indirect ELISA (Ab ELISA)

Blood samples without anticoagulant were allowed to clot and the serum was stored at -20 °C until required. The production of antibodies (Ab) against *Map* was determined by an indirect ELISA, using a protoplasmic antigen of *Map* (PPA-3; Allied Monitor Lab. Inc., Fayette, USA) and horseradish peroxidase conjugate protein G as a secondary Ab (Biorad, Hercules, USA). The technique was performed as previously described [23]. The absorbance values were measured spectrophotometrically at 450 nm using an ELX800 ELISA reader (Bio-Tek Instruments, Winooski, USA). The results were expressed as a quotient between the mean O.D. of each sample sera and the mean O.D. of the positive control serum in each plate. An animal is considered positive when this quotient is higher than 0.9 [8].

### Pathological studies

Complete necropsies were performed in all the animals. Gross examination was carried out, with special attention to the gut and related lymph nodes. Samples from the ileocecal valve (ICV), ileum (IL) (three 5-cm samples, taken 20, 40 and 60 cm from the ileocecal valve), jejunum (JJ) and jejunal Peyer’s patches (JPP) (at least 3 patches from each of the proximal, medium and distal zones), the caudal mesenteric lymph node (MLN), one jejunal lymph node (JLN) and ileocecal lymph nodes (ICLN) were taken for histopathological examination. All the tissues were fixed in 10% neutral buffered formalin, dehydrated through a graded alcohol series before being embedded in paraffin wax. Sections 4 μm thick were stained with haematoxylin and eosin (HE), Masson’s Trichrome to stain connective tissue and by the Ziehl-Neelsen (ZN) technique for acid-fast bacilli (AFB) detection.

Representative granulomatous lesions found in lambs from all the experimental groups were assessed immunohistochemically for the presence of *Map* or its antigens, using an EnVision + HRP visualization kit (Dako North America, Carpinteria, USA). The sections were incubated with a specific rabbit anti-*Map* serum at a dilution of 1/9000, as described elsewhere [28].

All the lesions consistent with *Map* infection observed in the digestive tract were classified following the guidelines previously proposed [5,28] for paratuberculosis lesions in the ovine species, according to the presence and location of granulomas in the different intestinal lymphoid tissue compartments.

After the conventional histopathological examination, the number of granulomas per tissue section was quantified in the following samples: ICV, 3 samples of the IL, JJ and JPP (proximal, middle and distal), MLN, JLN and ICLN. Three tissue sections were randomly selected from each intestinal site and 2 tissue sections from each lymph node, so that a total of 30 intestinal and 6 lymph node tissue sections were analysed from each animal. Sections were assessed blind and the mean number of granulomas per tissue section in each site was recorded by the same observer (MF), distinguishing those granulomas located in the lymphoid tissue from those in the associated lamina propria (LP) or in the mucosa not related to lymphoid tissue.

### Nested PCR

The detection of *Map* DNA was assessed using a nested PCR method that was performed from paraffin-embedded tissues. In total, 10 μm of ICV, middle JPP and JLN tissue sections adjacent to those used for the ZN and immunohistochemical studies were cut twice, and DNA was isolated using Speedtools Tissue DNA extraction kit according to the manufacturer’s instructions (Biotools® B&M Labs., Madrid, Spain). The nested PCR was carried out as previously described [27] using primers to detect the presence of *Map*-specific IS*900* DNA.

### Statistical analysis

Data on IFN- γ and Ab production as well as on granuloma count were subjected to analysis of variance using the general linear model procedure (GLM) of the SAS statistical package (version 9.1; SAS Institute, Cary, NC, USA) for the evaluation of treatment, time of killing and lesion location main effects and interactions. The results of the O.D. indexes obtained in the IGRA, Ab ELISA tests and the tissue granuloma count figures were logarithmically transformed to submit them to normal distribution-based tests of significance. Thus, differences among the experimental groups at each time of sampling or killing were evaluated using the Student’s *t*-test for pair-wise comparisons with the Tukey-Kramer correction for multiple comparisons, at the 95% significance level. The results of the IDT among the experimental groups at 120 and 360 dpi, and the PCR-positive frequencies were compared and tested for significance by chi-squared analysis.

## Results

One animal from the K-10 group died at 30 dpi, during the course of the experiment from causes unrelated to Johne’s disease (bacterial pneumonia) and was excluded from this study.

### Assessment of the colony forming units (CFU)

**Figure 1:**
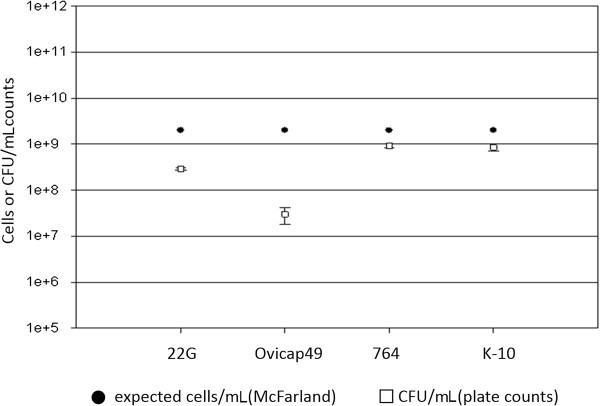
**Assessment of the bacterial load of the inocula.** Differences between the bacterial load of inocula used for the challenge of animals as assessed by optical density (McFarland) and CFU counts (plate colony counting).

The number of CFU obtained after the culture of the first and fourth (shown in Figure 1) aliquots of the different inocula gave similar results. As shown in Figure 1, the difference between the expected number of cells/mL as assessed by McFarland readings and CFU/mL counts as assessed by plating serial dilutions of inocula was lower than one logarithm in all cases except for the Ovicap49 strain. In this case the mean value for CFU/mL counts was only 3 × 10^7^ (standard deviation = 1.41 × 10^7^).

### IFN-γ test (IGRA)

**Figure 2:**
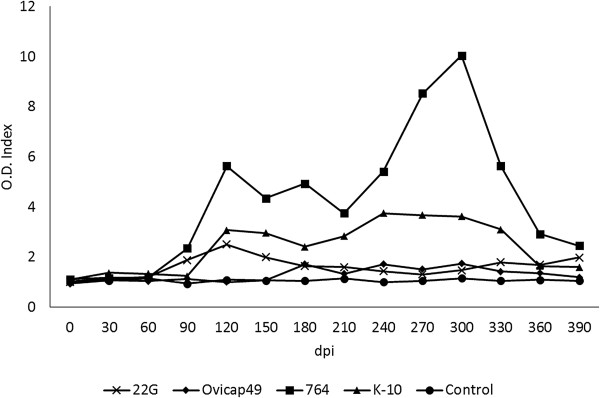
**Kinetics of the specific IFN-γ release by whole blood stimulated with avian PPD in the five experimental groups.** The results are expressed as an index obtained after dividing the mean O.D. of each sample stimulated with avian PPD by the mean O.D. of the same sample incubated with PBS. From 0 to 150 dpi, each group was formed by 6 lambs, while from 180 to 390 dpi they were composed of 4 animals (except group K-10, formed by 5 and 3 respectively). dpi: days post infection.

Figure 2 shows the IFN-γ production in the different experimental groups. No significant differences were observed between the groups infected with an S-type strain and the control group, although sporadically some of the infected lambs showed an index value considered as positive (> 2). However, in both experimental groups challenged with the C-type strains, the IFN-γ production was significantly higher than in the control or S strains-infected groups (*P* < 0.05) between 120 and 330 dpi. The highest values were mainly reached between 240 and 330 dpi, with significant differences (*P* < 0.05) between 764 and K-10 groups. At 120 dpi (Figure 2), both C-type strain-infected groups showed the earliest significant increase, that was also higher in the 764 group (*P* < 0.05).

### Intradermal skin test (IDT)

**Figure 3:**
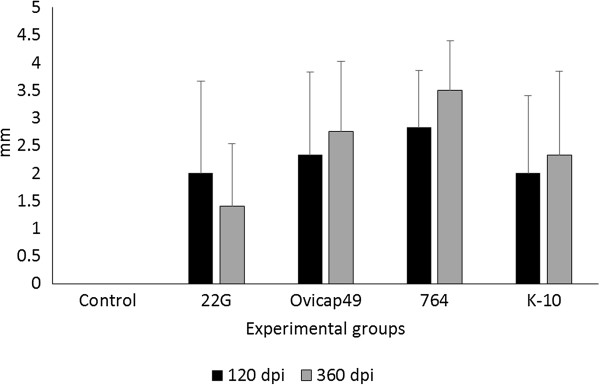
**Response to the intradermal skin test (IDT).** The results are expressed as the skin-fold thickness increase (in mm) after intradermal injection of avian PPD, at 120 and 360 dpi in the five experimental groups. Error bars: standard deviation.

All the infected groups showed a significant increase in skin thickness (*P* < 0.05) compared to the control group, both at 120 and 360 dpi (Figure 3), except for lambs infected with the 22G strain at 360 dpi. No significant differences in this response were detected among the different groups either at 120 or 350 dpi.

### Indirect ELISA

**Figure 4:**
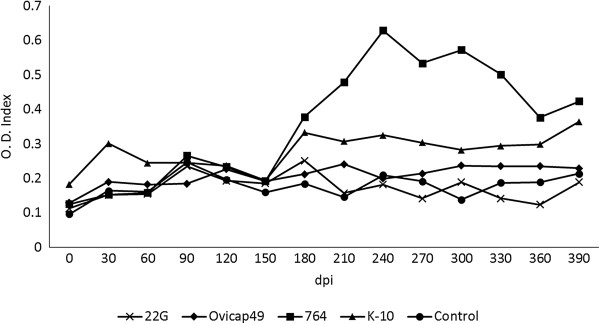
**Kinetics of the antibody production of the five experimental groups, assessed by indirect ELISA.** The results are expressed as an index obtained after dividing the mean O.D. of each sample by the mean O.D. of the positive control from each plate. From 0 to 150 dpi, each group was formed by 6 lambs, while from 180 to 390 dpi they were composed of 4 animals (except group K-10, formed by 5 and 3 respectively). dpi: days post infection.

The Ab production in the different experimental groups throughout the experiment is shown in Figure 4. No significant differences were observed between the groups, except for a significant increase (*P* < 0.05) between 210 and 330 dpi in the Ab levels of the animals from the 764 group.

### Pathological findings

#### Gross lesions

**Figure 5:**
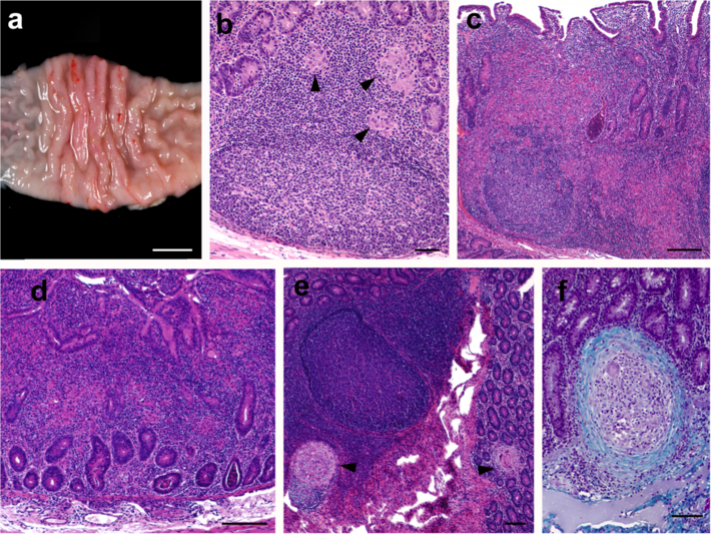
**Pathological findings in the experimentally infected lambs. (a)** Focal thickening of the jejunal mucosa in a lamb from the 764 group euthanized at 150 dpi. Bar = 1 cm. **(b)***Focal* lesion composed of a group of macrophages (arrowheads) seen in the interfollicular area of the JPP, observed in a lamb from group Ovicap49 culled at 390 dpi. HE. Bar = 50 μm. **(c)** Severe granulomatous infiltrate invading and modifying the lymphoid tissue architecture, that is also present in the related LP. JPP. Lamb from K-10 group with a *multifocal b* lesion, culled at 150 dpi. HE. Bar = 500 μm. **(d)***Diffuse* lesion characterized by the thickening of the jejunal lamina propria with enlargement of the intestinal villi due to the presence of an infiltrate formed by several poorly-defined granulomas surrounded by numerous lymphocytes. Jejunum. Lamb from 764 group euthanized at 150 dpi. HE. Bar = 350 μm. **(e)** Two small and very well demarcated granulomas (arrowheads) present in the lymphoid tissue and adjacent lamina propria (*multifocal a* lesion) that do not significantly alter intestinal morphology. JPP. Animal from 764 group culled at 390 dpi. HE. Bar = 200 μm. **(f)** Detail of a “regressive” type granuloma, with a thick fibrous capsule surrounding the macrophages and giant cells, seen in the jejunal lamina propria in a lamb from 764 group culled at 390 dpi. Masson’s Trichrome. Bar = 70 μm.

In the two lambs euthanized at 150 dpi from group 764, one and two areas of thickening of the intestinal mucosa, between 1–2.5 cm long, were observed respectively in the middle part of the jejunum (Figure 5a). No other gross changes related to paratuberculosis were seen in any of the rest of the lambs regardless of the time of sampling.

#### Microscopic lesions

Granulomatous lesions consistent with *Map* infection were found in the tissue sections from all infected lambs euthanized at 150 dpi, regardless of the group, and in all but three animals (one from 22G, Ovicap49 and K-10 group each respectively) examined at 390 dpi. However, the type of lesion varied between the S and C-type *Map* strain infected groups. To categorize each animal, the final classification was always based on its more severe intestinal granulomatous lesion (Table 2).

**Table 2:** Distribution of experimental animals in the infected groups according to their lesion type and the results of the nested-PCR

				**Nested PCR results**		
**Experimental group**	**Time of sampling**	**Animal ID**	**Type of lesion**	**ICV**	**mJJP**	**JLN**
**S-type strains**						
**22-G**	150 dpi	1	Focal	+	+	+
		2	Focal	+	+	+
	390 dpi	3	Focal	+	+	+
		4	Focal	+	+	-
		5	Focal	+	+	+
		6	No lesion	-	+	-
**Ovicap 49**	150 dpi	7	Focal	+	+	+
		8	Focal	+	+	-
	390 dpi	9	Focal	+	+	+
		10	No lesion	+	-	-
		11	Focal	-	+	-
		12	Focal	+	-	-
**C-type strains**						
**764**	150 dpi	13	Diffuse	+	+	+
		14	Diffuse	+	+	+
	390 dpi	15	Multifocal a	+	+	+
		16	Multifocal a	+	+	+
		17	Focal	-	+	-
		18	Multifocal b	+	+	+
**K-10**	150 dpi	19	Multifocal b	+	+	+
		20	Multifocal a	+	+	+
	390 dpi	21	No lesion	-	-	-
		22	Multifocal a	+	+	+
		23	Focal	-	+	+

Lambs from S strain infected groups (both 22G and Ovicap49) showed lesions that were categorized as *focal.* They were formed by small, well-defined granulomas composed of groups of 20–50 macrophages with a large pale cytoplasm and large nuclei with a few lymphocytes scattered among them (Figure 5b). These granulomas were found exclusively in the interfollicular area of the intestinal lymphoid tissue, either in the ileocecal valve or the jejunal Peyer’s patches (Figure 5b). The presence of these granulomas, due to their small size, did not alter the normal structure of the Peyer’s patches. These focal lesions appeared in the four lambs euthanized at 150 dpi, and in 6 out of the 8 (75%) lambs analysed at 390 dpi. The remaining two animals (one from each experimental group) did not show lesions related to *Map* infection.

Differences in the lesion type between the groups infected with the S and C strains were observed. At 150 dpi, among lambs from the K-10 group, one animal had lesions categorized as *multifocal a,* composed of more numerous granulomas than those seen in the focal forms. In addition to the granulomas present in the interfollicular areas of the Peyer’s patches, small and well-defined granulomatous lesions were also detected in the lamina propria (LP) related to the intestinal lymphoid tissue. The morphology of the villi was not substantially modified. In the remaining lamb from the K-10 group, the lesion was classified as *multifocal b*. In that animal, granulomatous changes were more severe in the lymphoid tissue and related LP (Figure 5c), but were also extended to the LP not associated with the lymphoid tissue. At this location, granulomas were seen between the intestinal glands and caused a focal thickening of the LP, without a clear disruption of the normal structure. In both lambs from group 764 culled at 150 dpi, lesions were classified as *diffuse.* They were composed of large numbers of granulomas that coalesced and invaded the complete structure of the Peyer’s patches, causing a clear modification of their normal architecture. Poorly demarcated granulomatous lesions were also found in the LP related and non-related to the lymphoid tissue (Figure 5d). In areas of the jejunum, the intestinal mucosa was markedly thickened due to the presence of granulomas surrounded by a large number of lymphocytes (Figure 5d). The presence of this infiltrate caused the enlargement and fusion of the intestinal villi and the separation of the intestinal glands. Occasionally cell debris was observed in the lumen of the glands (Figure 5c, d).

A constant feature in both groups of lambs infected with the C-type strains, in contrast to those infected with the S strains, regardless of the lesion type, was the occasional finding of multinucleated Langhans giant cells among the cells forming the granulomas, and the sporadic presence of central areas of caseous necrosis exclusively in some of the granulomas located in the lymphoid tissue, especially in the largest ones.

Among the three lambs from the K-10 group euthanized at 390 dpi, one of them had *focal* lesions, another had a *multifocal a* type lesion and there were no lesions in the last. In lambs from group 764, no diffuse lesions were observed at 390 dpi, in contrast to the pathological findings at 150 dpi. One animal had a *multifocal b* lesion, two had *multifocal a* lesions (Figure 5e), and a *focal* form was detected in the remaining lamb. The morphological features of the granulomatous lesions found at 390 dpi among the animals infected with the C-type strains, regardless of the group, differed from those observed at 150 dpi. In the former, all the granulomas, both those located in the lymphoid tissue area and in the LP (related or unrelated to the lymphoid tissue) were significantly smaller, round and very well-demarcated from the adjacent tissue by a fibrous tissue capsule than those found at 150 dpi (Figure 5e). When the sections stained with Masson’s trichrome were examined, a marked amount of collagen fibres was seen among the macrophages, lymphocytes and Langhans giant cells that formed the granulomas, regardless of their location in the intestine (Figure 5f). Very occasionally, granulomas found in the lymphoid tissue in the lamb from group 764 with a *multifocal b* lesion, showed a necrotic centre with dystrophic mineralization.

Besides the intestine, granulomatous lesions were also found in the lymph nodes, mainly in the MLN and JLN. In lambs from K-10 and 764 groups showing the more severe intestinal lesions (*multifocal b* and *diffuse*), a diffuse granulomatous lymphadenitis was observed. It was composed of a granulomatous infiltrate, similar to that seen in the intestine, formed by macrophages and some Langhans giant cells, that was spread throughout the interfollicular and paracortical areas, with a multifocal distribution. In a number of occasions, always corresponding to the animals with *diffuse* lesions, these granulomas coalesced and invaded the adjacent lymphoid follicles, causing a marked distortion in the morphology and enlargement on the lymph node. A necrotic centre, occasionally with mineralization, was also seen in some granulomas. On the contrary, the lambs with *focal* or *multifocal a* lesions in the intestine from any of the four experimental groups, showed a focal granulomatous lymphadenitis characterized by one or two small granulomas composed of less than 10 macrophages (rarely small multinucleated cells were detected in lambs from groups K-10 and 764), located in the interfollicular part of the cortical area of the ICLN, MLN or JLN.

No AFB or *Map* antigens were detected in any of the control lambs or in all the lesions classified as *focal* or *multifocal a*, regardless of the experimental group. Only in animals with *multifocal b* or *diffuse* lesions were solitary or few AFB detected both by ZN and immunohistochemistry, only in the granulomas located in the LP. In the lymph nodes, occasional AFB were observed in samples of lambs with intestinal *diffuse* lesions.

### Granuloma count

Table 3 shows the mean of the total granuloma counts per animal, corresponding to the different experimental groups and the time of euthanasia. Differences in the lesion severity, with variations among individuals of the same group, were observed. Significant reduction in the number of granulomas (*P* < 0.001) was noticed between animals euthanized at 150 and 390 dpi in both groups infected with C-type strains whereas no differences were found in animals infected with the S-type strains either between 22G and Ovicap49 groups or the time of sampling. Moreover, animals from the 764 group had a higher amount of granulomatous lesions, both at 150 and 390 dpi (*P* < 0.001) than lambs from the K-10 group.

**Table 3:** Total granuloma counts per animal

	**150 dpi**	**390 dpi**
**S-type strains**		
22G	2.705 ± 0.86	2.16 ± 1.57
Ovicap49	2.42 ± 1.04	1.63 ± 1.75
**C-type strains**		
764	144.355 ± 38.4	27.122 ± 14.5
K-10	22.305 ± 2.18	8.21 ± 12.4

Granuloma counts from the tissues of lambs infected with the S and C strains and euthanised at 150 and 390 dpi. Mean number of granulomas per animal ± standard deviation.

**Figure 6:**
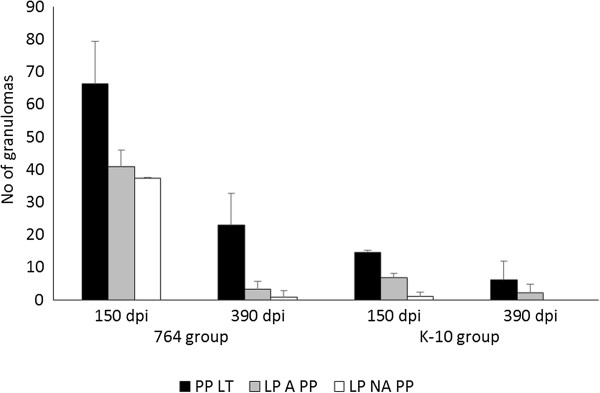
**Granuloma counts from the C-type strains infected lambs in the different intestinal compartments.** Count of granulomas from the tissues of the C-type strains infected groups (764 and K-10) culled at 150 and 390 dpi. Mean number of the granulomas per animal, corresponding to their location in the different intestinal compartments. dpi: days post infection. Error bars: standard deviation. PP LT: Peyer’s patches lymphoid tissue. LP A PP: lamina propria associated with Peyer’s patches. LP NA PP: lamina propria not associated with Peyer’s patches.

Figure 6 shows the mean granuloma counts corresponding to the different intestinal compartments examined in lambs from K-10 and 764 groups at 150 and 390 dpi. A reduction in the number of granulomas present in the lymphoid tissue and related LP between 150 and 390 dpi (*P* < 0.05) was observed in both groups. It is worth highlighting the marked decrease in the number of granulomas located in the LP not associated with the lymphoid tissue in the 764 group (*P* < 0.001), in agreement with the previously described types of lesion. In the groups infected with the S-type strains, all the granulomas appeared exclusively in the intestinal lymphoid tissue, with no differences between groups or time of sampling (Table 3).

**Figure 7:**
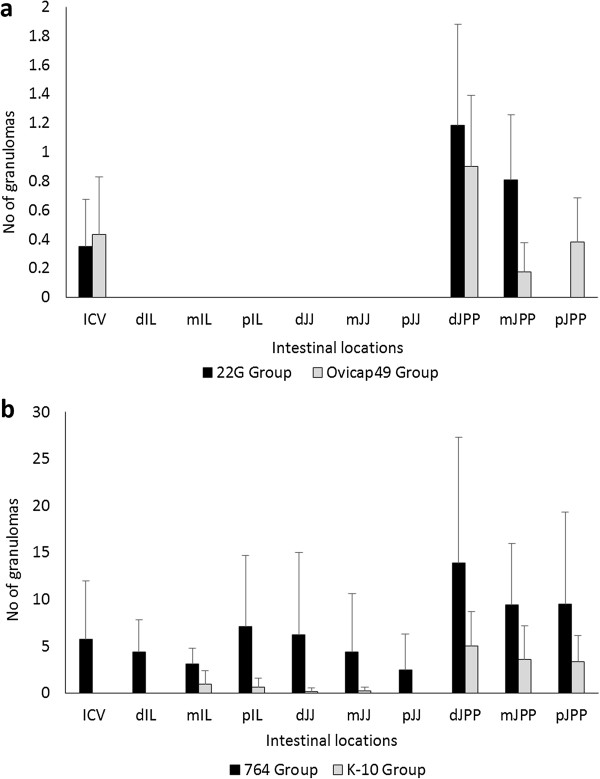
**Granuloma counts according to their intestinal location.** Total granuloma count from the tissues of the S **(a)** and C **(b)** strains infected groups, regardless of the time of sampling. Mean of the total number of granulomas per tissue section and animal, according to their intestinal location. ICV, ileocecal valve; IL, ileum; JJ, jejunum; JPP, jejunal Peyer’s patches. d, distal; m, medium; p, proximal. Error bars: standard deviation.

The distribution of the lesions in each intestinal location examined in the different experimental groups is shown in Figure 7. In the groups infected with S-type strains (Figure 7a), lesions were found exclusively in the ICV and the different JPP. It is noteworthy that IL samples were always negative, even though lymphoid tissue was present in all the sections examined. Although granulomas appear to be slightly more numerous in the JPP samples than in the ICV in both groups (Figure 7), differences were not statistically significant. In contrast, in the groups infected with C-type strains (Figure 7b) granulomas were appreciated in all the samples with lymphoid tissue (ICV, IL and JPP). Although the number of granulomas seemed to be higher in the JPP than in the other regions, differences were only statistically significant (*P <* 0.05) in animals from group K-10. When comparing both groups, granuloma counts were higher in all the locations in the 764 than in the K-10 group, remarkably in the ICV, IL and JJ samples (*P* < 0.001).

In addition to the intestine, granulomas were also noted in the associated lymph nodes. They were always more numerous in animals infected with the C- rather than the S-type strains (*P* < 0.05). When considering the different groups, no differences were found between the S strain challenged groups, but granulomas were more numerous in the 764 than in the K-10 group (*P* < 0.05). In accordance with the results of the intestine, a significant decrease was also appreciated in the granuloma counts between 150 and 390 dpi (*P* < 0.05) in animals from both groups infected with the C-type strains. Granulomas were counted more frequently in the JLN than in the other lymph nodes in all the groups.

### Nested PCR

IS*900 Map*-specific sequence was demonstrated by nested PCR in all 12 lambs infected with the S-type strains including animals with no lesions, from at least 1 of the 3 analysed tissue sections (Table 2). No significant differences in the positivity detected among the examined locations were observed. One lamb euthanized at 390 dpi from the K-10 group, which was categorized as having no lesions, was negative to the presence of *Map* DNA (Table 2). The other animals infected with C-type strains were positive from at least 1 tissue section examined. As in the rest of the groups, no statistical differences were seen in the positivity rates among the locations analysed.

All tissue sections from the uninfected control lambs were negative using PCR.

## Discussion

The results of this study show that sheep can become experimentally infected with either C or S-type strains of *Map*, as previously documented [10,23], but clear differences in the immune response and especially in the lesion development occur in relation with the type of strain.

Variations in the morphology of the granulomatous lesions due to different *Map* isolates were previously reported [23], where infection with C-type strains caused the appearance of a larger number of giant cells and central caseous necrosis in lambs examined at 150 dpi, which was demonstrated in the current study. These findings are closer to the lesions described in natural bovine paratuberculosis [6] than those seen more frequently in sheep, where giant cells are not a very characteristic hallmark [1,5]. Considering that our study and that of Verna et al. [23] used different C-type strains, with similar pathological findings, it seems feasible to conclude that C strains rather than host species, are the cause of some of the pathological characteristics of *Map* infection.

Some of the lambs infected with C-type strains, at 150 dpi showed diffuse lesions affecting wide areas of intestinal mucosa not associated with the lymphoid tissue, which has been regarded as related to more advanced stages of *Map* infection [4-6,23,28,29]. According to the pathological findings at 390 dpi, lesions induced by C strains in sheep showed an evolution towards resolution, with a marked decrease in their severity, characterized by lower numbers of smaller, well-encapsulated and fibrotic granulomas. Although Verna et al. [23] noted some degree of fibrosis in some lesions induced by C-type strains, their study ended at 150 dpi and moreover, lesion severity was not assessed. Granulomas showing fibrosis could be considered as “regressive” type lesions, as similar histological features have been described in lambs vaccinated and experimentally infected with *Map*[4,29,30], where they have been associated with a regression of the infection in immunized animals, or in minipigs infected with *Mycobacterium tuberculosis* in the lung [31], related to a contention of the infection. This finding would suggest that in lambs infected with C-type strains, if the experimental infection had been prolonged for a longer period, infection could have been contained or even regress, with resolution of lesions and recovery of tissue morphology. The fact that one lamb from the K-10 group that showed a specific cellular immune response did not show any lesion and was negative for *Map* DNA identification in the tissues sampled at 390 dpi, further supports this hypothesis.

In paratuberculosis vaccinated animals, the efficacy of the vaccine in controlling the progression of the infection has been associated with the induction of a protective and vigorous cellular immune response [30,32]. In this study, the intense IFN-γ production seen in C-type strains infected lambs could be related to the regression of the lesions. In a previous experiment, S-type *Map* isolates also proved to be less pathogenic and induced a weaker cell-mediated immune response compared with a C strain, when infecting sheep [10], but a pathological evaluation of the lesions was not assessed. Thus, from our results, it can be hypothesized that infection induced by C-type strains could have reached its highest development around 150 dpi, but infected lambs were able to mount a more efficient and specialized immunologic response that would have caused a regression of the lesions. A similar mechanism has been proposed to occur in adult ewes or cows infected with *Map* showing a higher resistance to infection [27,28,33]. It might have been interesting to have followed these animals over a more uniform and longer timescale such as culling every two months after infection for a longer period.

On the contrary, lambs infected with S-type strains only showed focal granulomatous lesions, restricted to the lymphoid tissue, and not associated with a detectable peripheral immune response. This type of lesion has been described in experimental paratuberculosis in sheep [4,28,29,34] and also in adult animals in natural cases, raising the hypothesis that they could be considered as latent lesions present in adult animals infected earlier in life or as initial lesions of recently infected adult individuals [5]. Our results, showing the presence of similar focal granulomatous lesions at 150 dpi and 390 dpi in the infected animals that did not appear in the control lambs, would further support the hypothesis of the latent character of the focal lesions.

In natural or experimental cases, the majority of sheep with focal lesions show a marked peripheral cellular immune response [9,27]. However, in our study, only some lambs were sporadically positive to IGRA, with no statistical differences when compared with the control animals when taking the overall results, even though a specific but weak cellular immune response was detected by IDT. This finding would indicate that IDT may be more sensitive than IGRA, as has been previously reported [9,35]. However, this is not in agreement with the results obtained in the groups infected with C-type strains, where the IDT response was similar to the S groups but accompanied by a higher IFN-γ response. From these results, it can be hypothesised that there are differences in the immune response induced by both types of strains, as has been demonstrated in several in vitro studies [20,21]. Moreover, the hypothesis that IDT and IGRA would measure different factors operating in the specific cell mediated immune response against *Map* should be considered and further investigated.

The possibility that these animals with focal forms have not mounted a persistent and measurable peripheral specific production of IFN-γ should also be considered, in the light of the work of Vazquez et al. [36], where they found a large proportion of cattle with focal lesions negative to IGRA. In the present study, lambs infected with S-type strains would fit the recently proposed pathogenesis model of *Map* infection [36] that considers focal lesions as a condition of certain natural resistance or premonition sustained by the presence of a continuous confined inflammatory focus. Furthermore, our results from the peripheral immune responses in the whole experiment, did not support the standard model of immunity to paratuberculosis in which in the early stages of the disease peripheral IFN-γ release is the main response, while it decreases in the more advanced forms where humoral responses predominate [2,37]. Our results in the lambs infected with C-type strains show a mixed cellular and humoral response from 120 to 330 dpi, probably in coincidence with the highest severity of the inflammatory response, while in the lambs challenged with S-type strains, there were minimal inflammatory lesions, and no IFN-γ production was observed. This finding would coincide with recent studies [10,36,38] that question the Th1 dominancy in the early stages of *Map* infection.

Recent work carried out after the in vitro infection of bovine macrophages with different *Map* strains have shown different patterns of cytokine expression: while C-type strains have a high rate of survival inside the cells related to an anti-inflammatory response characterized by an up-regulation of IL-10, S-type strains showed a lower persistence with a significantly up-regulated pro-inflammatory response [20,21]. These findings indicate that the survival of the *Map* strains within bovine macrophages is strongly associated with the specific host from which the isolates were initially isolated [20]. A similar mechanism could operate at the level of the intestinal macrophages after *Map* infection, suggesting that the local immune responses occurring at the intestinal lymphoid tissue level should play an important role in *Map* pathogenesis. This deserves to be further investigated in the lambs from this study. In this sense, previous studies [3,39] have shown the importance of the immune response mounted in the Peyer’s patches in the early stages of paratuberculosis and its lack of correspondence with the peripheral immune response.

In contrast to other experimental infections carried out by our group in lambs using S-type strains [23,28], none of the lambs of this study showed widespread lesions, that in previous studies were seen as soon as 120–150 dpi. It could be postulated that the use of low passage, pure culture of *Map* inocula in this experiment instead of an intestinal mucosal homogenate from a naturally affected sheep, as in the other studies, could explain this. *Map* infections are more easily and rapidly established when the challenge inoculum was prepared from gut mucosal tissue than from cultured bacteria [10,40]. Our results possibly mimic what occurs in natural conditions, where a great majority of infected animals show focal and latent lesions [5,36] similar to those found in our study. Further supporting this finding, in the experiment carried out by Begg et al. [34] infecting larger groups of lambs with a cultured S strain, animals with widespread lesions only appeared in some lambs examined 10–19 months after the infection. Thus, it seems feasible that, should the length of the experimental infection had been prolonged, lesions could have progressed towards more severe forms in some of the lambs infected with the S *Map* strains. On the contrary, the possibility that the S-type strains used had a lower pathogenicity than those used in previous studies cannot be discounted. The existence of variations in pathogenicity among the different strains of *Map* has already been reported [13,23,40] and has been confirmed among the C strains in this study, where the 764 isolate induced more intense immune and pathological responses than the K-10 strain. The fact that the latter is a *Map* reference strain with a high number of culture passages could have contributed to its lower pathogenicity [40]. However, no differences were observed between the two S strains employed; both were low passage cultures, isolated from clinical cases of ovine paratuberculosis. Although traditionally ovine pigmented strains have been considered as having a higher pathogenicity [13,41], this fact is not supported by our results.

Differences in the pathogenicity have also been associated with the dose administered to the experimental animals [4,28]. In our study, the dose employed is comparable to those used in other studies [10,23,28,34] and has been shown to cause the infection. A good correlation was observed between the expected number of bacteria assessed by the McFarland method and the CFU counts evaluated by plating serial dilutions, as previously stated [26], except for the pigmented strain Ovicap49. Such an unexpected difference between optical density and viable cell count could be explained by the enormous difficulty in culturing this concrete ovine pigmented strain, especially on the surface of solid media, rather than by an actual reduction in the number of viable units.

This study has also shown the importance of the intestinal lymphoid tissue in the establishment of paratuberculosis infection, regardless of the strain used, since most of the granulomatous lesions appeared in this tissue, as was previously observed [4,5,23,28,29], further confirming the role of the Peyer’s patches both as a primary portal of entry of *Map* in the organism and in the persistence for longer periods of time of focal granulomatous lesions representing forms of latency or resistance.

Regarding the distribution of the lesions along the different intestinal sites and their intensity, the granuloma count showed a marked variation between the individuals and the different sections of the intestine, as has previously been reported as a typical feature of natural and experimental paratuberculosis [4,28,34,40]. In all the experimental groups, JPP has been the region harbouring the highest amount of lesions followed by the ICV, whereas lesions in the ileal Peyer’s patches (IPP) appeared in low numbers and only in lambs infected with the C-type strains. This finding has been reported previously [28,42] and could indicate a different functional pattern between IPP and JPP related to the morphological and lymphocyte distribution differences observed [42,43]. Lesions only appeared in the lymph nodes when they were already present in the intestine in a remarkably lower number. This finding, supported by other studies [5,28], would confirm the critical role of the intestinal lymphoid tissue in the start of *Map* infection, which would be detected in the lymph nodes only when lesions are well established in the gut.

The recognized difficulty in culturing *Map* from ovine tissues [19,29] in terms of slowness and low performance of bacterial isolation, was the reason for using a nested PCR method for confirming *Map* infection in the tissues of the challenged animals, in addition to the presence of the specific granulomatous lesions or AFB. This method has successfully been used and shown to have a higher sensitivity than *Map* culture or other PCR methods [28,44]. However, it has to be taken into account that the PCR method identifies DNA in the tissues that could have originated from non-viable and/or degraded bacilli. In our study, the presence of *Map* DNA was demonstrated in all infected animals, except one from the K-10 group culled at 390 dpi in which no lesion was detected, with no other differences with the time of sampling, in contrast to the lower number of granulomas detected at 390 dpi. It can be hypothesised that if bacterial culture had been performed, a lower number of colonies would have been isolated at 390 dpi in C strain infected lambs. Furthermore, similar to previous studies [28], two lambs infected with S-type strains, in which no lesions were observed, were also positive for PCR. In contrast to the higher prevalence of lesions in the JPP, no differences in the percentages of positivity by PCR were detected among the three regions of gut analysed, suggesting that the DNA from *Map* is spread along the intestinal tissues even without being related to evident lesions.

The low rate of identification of AFB in the tissues either by ZN or immunohistochemical methods was not surprising. Previous studies have shown that the focal and multifocal lesions contain no or very few AFB [5,6,23,28]. The morphology of the diffuse lesions found in the 764 group was consistent with the so-called “lymphocytic” diffuse forms of paratuberculosis [1,5,6], characterised by the presence of very low numbers of bacteria.

This study has shown clear differences in the pathogenesis of *Map* infection related to the type of strain used in an experimental infection of lambs. Infection caused by C-type strains was more rapidly and easily established, showing the most severe lesions and a stronger immune response. However, as the infection progresses, a marked reduction in the amount and severity of the lesions, consistent with a regressive character, was observed in association with a high peripheral cellular response suggesting that, with time, infection could have disappeared in these animals. In contrast, lambs infected with S-type strains developed focal granulomas located in the intestinal lymphoid tissue that persisted throughout the experiment. Although, in field cases, cross infection between S and C strains has been reported [17-19], its occurrence has been considered as infrequent [14,15,45]. Considering our results, it seems feasible that in areas where cattle and sheep graze together and cross infection could occur naturally [18,46], most of the sheep infected with C-type-strains could recover from the infection.

## Competing interests

The authors declare that they have no competing interests.

## Authors’ contributions

MF performed the experiment, participated in all the immune response and pathological studies and collaborated in the analysis of the data and writing of the paper. JB contributed to the sample collection, pathological studies, interpretation of the results and helped to draft the manuscript. IAS prepared the administered inocula and collaborated in writing the paper. MF participated in the sample collection, pathological studies and carried out the immunohistochemical analysis. PC performed the immune response analysis and helped in the sample collection. LD contributed to sample collection and to the pathological studies. JFGM collaborated in the pathological studies. JMG participated in the design of the experiment and inocula preparation. MCF collaborated in the sample collection, analysis of the data and helped write the manuscript. VP conceived and designed the experiment, analysed the data and wrote the paper. All the authors read and approved the final manuscript.
